# Ultra-old patients and long-term survival after hip fracture: a real-world assessment

**DOI:** 10.3389/fmed.2023.1200007

**Published:** 2023-07-24

**Authors:** Debora Tiso, Monica Pizzonia, Chiara Giannotti, Luca Tagliafico, Alessio Signori, Alessio Nencioni, Fiammetta Monacelli

**Affiliations:** ^1^Geriatrics Clinic, Department of Internal Medicine and Medical Specialties (DIMI), University of Genoa, Genoa, Italy; ^2^IRCCS Policlinico San Martino Hospital, Genoa, Italy; ^3^DISSAL, Department of Health Science, University of Genoa, Genoa, Italy

**Keywords:** hip fracture, ultra-old, frailty, overall mortality, prognostic score

## Abstract

It’s still undetermined whether ultra-old persons, aged >90 years, are able to tolerate hip fracture surgical stress while maintaining their functional reserve, and even fewer studies have investigated the role of frailty on the risk of mortality, disability, or morbidity in the ultra-old. This is a prospective study performed at the Orthogeriatrics Ward of the IRCCS Policlinico San Martino (Genoa, Italy) that consecutively enrolled 205 older adult patients with hip fractures due to low-energy trauma. Namely, 85 patients were categorized as ultra-old, and 120 patients (64–89 years) were the younger control group. Demographic data, perioperative data, and rehabilitation data were collected. Here we estimated the overall survival and related predictive variables in hospitalized ultra-old hip fracture patients based on a methodologically robust frailty stratification (Rockwood 40-item tool). The median OS for the ultra-old was 18.7 months, which also showed a doubled 1-year mortality risk. Our findings assessed that frailty in the presence of malnutrition, delayed verticalization, and post-operative respiratory complications was associated with a two-fold increase in the risk of long-term mortality, irrespective of advanced chronological age in the ultra-old. Although the higher mortality rate in these patients may be related to *a priori* lower life expectancy, chronological age alone is an insufficient prognostic determinant for unfavorable outcomes. Our multicomponent prognostic score can be used in combination to stratify frailty in the ultra-old for timely screening and to deliver goals of care discussions prior to surgery, potentially targeting new orthogeriatric pathways for the improvement of appropriateness and treatment intensity.

## Introduction

The number of persons aged 90 years and older (ultra-old) is estimated to increase four-fold between 2010 and 2050 (1), and very advanced age has often been deemed a correlate for multimorbidity, frailty, and disability. In particular, a key consideration for surgical management in the ultra-old is the ability of these patients to tolerate surgical stress while maintaining their functional reserve and resilience. Similarly, fragility fracture is a common geriatric syndrome and has a major impact on overall survival, affecting functional status, disability, quality of life, and overall survival (2).

Some studies indicate that ultra-old patients have the poorest outcomes after hip fracture surgery (3, 4), suggesting that these patients have increased 30-day mortality rates compared to their younger counterparts (5). However, there is a paucity of studies on how hip surgical stress impacts functional recovery in this extreme age group, and even less research has investigated the role of frailty on the risk of mortality, disability, or morbidity after hip surgery in the ultra-old (6). To date, there are several inflated risks and clinical confounders, such as inadequate stratification by age and frailty, which may lead to inherently inconclusive remarks in this ultra-old population. Therefore, it is still undetermined whether chronological age alone is a reliable predictor of 1-year mortality and functional decline, or whether the co-occurrence of frailty or comorbidity may predispose these patients to worse clinical outcomes (7, 8).

Based on this background, we aimed to conduct a prospective study to assess the overall survival (OS) in a matched cohort of surgically treated ultra-old hip fracture patients compared to their younger counterparts and to assess the main clinical variables associated with the long-term outcome.

## Methods

This is a prospective, single-center study performed at the Orthogeriatrics Ward of the IRCCS Policlinico San Martino (Genoa, Italy) from September 2019 to November 2021, which consecutively enrolled 205 hospitalized patients with hip fractures.

Demographic data and postoperative complications, including anemia, delirium, acute renal failure, urinary tract infection, respiratory complications, acute cardiac failure, bed sores, and days of verticalization, were collected.

Eligible patients were ≥ 65 years of age and had sustained hip fractures due to low-energy trauma (fragility fractures, lateral peritrochanteric hip fractures, and medial hip fractures). Patients were excluded if informed consent was lacking, surgery was precluded due to surgical or clinical instability, a high-energy trauma was involved, or fractures were pathologic or periprosthetic in nature. In total, 50% of the patients sustained lateral peritrochanteric hip fractures treated with intramedullary nailing procedures (Endovis BA; Citieffe, Bologna, Italy). The other half had sustained medial hip fractures that were treated with arthroplasty. All patients received in-hospital peri- and post-operative multidisciplinary orthogeriatric care (i.e., two expert geriatricians, and orthopedic staff members) (9, 10). Time to surgery (<48 h) and early 24-h verticalization after surgery were the primary rehabilitation goals to be achieved.

Overall survival after hospital discharge (censoring date of 20 January 2022) was also collected through the ASL3 electronic database of the Province of Genoa, Italy, as was 1-year mortality.

Each patient received a Comprehensive Geriatric Assessment (CGA), including a Handgrip test, HG, to assess sarcopenia; the Barthel index, and Instrumental Activities of Daily Living, IADL, to assess functional status; a Mini Nutritional Assessment, MNA-SF, to assess malnutrition; a Cumulative Illness Rating Scale-Geriatric, a CIRS, to assess multimorbidity; and a Delirium (4AT) test to assess delirium (Supplementary material S1). Polypharmacy was also collected.

Frailty status was also assessed with the Frailty Index according to the Rockwood 40-item tool (FI-40 item).

### Statistical analysis

Results were reported as median with interquartile range (IQR) or range for continuous variables and as absolute and relative frequencies for categorical variables.

The two groups of patients were compared on the basis of demographic and clinical variables using the Mann–Whitney test for continuous variables and Fisher’s exact test or chi-squared test for binary or categorical variables.

Univariable and multivariable Cox regression analyses were performed to assess the association of each clinical variable with OS. An interaction test was performed to evaluate differences in the association between the ultra-old group and the 65–84 year control group and OS for each clinical variable.

A multivariable analysis was performed for OS in the ultra-old age group, selecting for the model all clinical variables (*p* < 0.05) that were significant in the univariable analysis. Variables with a *p*-value >0.10 after inclusion in the model were removed from the final model. Parameter estimation from the Cox model to create the prognostic score was internally validated using the bootstrap approach with 500 replications. In the development of the prognostic score, MNA-SF was categorized at the cut-off of 8 and assessed with the survival ROC curve. The prognostic score was calculated using the regression coefficient-based (Schneeweiss) scoring system, where the weight assigned to each factor in the score was defined based on the regression coefficient obtained from the Cox regression model [Mehta et al. (11)].

The same statistical method, including the univariable and multivariable logistic regression models, was used when considering 1-year mortality risk as the primary outcome.

The prognostic score was stratified into three risk strata according to the likelihood ratio test, and after checking the survival estimates of the score. Analyses were conducted using Stata (v.16; StataCorp).

## Results

Of the 205 patients enrolled, 85 were aged 90 years or older (ultra-old), and 120 patients aged 64–89 years were considered the younger control group.

### Clinical phenotype of ultra-old patients

The clinical phenotype of the patients is illustrated in Table 1. Namely, 85 (41.5%) of 205 patients were categorized as ultra-old (median age 94 years, IQR 90–103), compared to the control group of 120 patients (58.5%), whose median age was 83 years (IQR 64–89).

**Table 1:** Clinical phenotype of patients.

Clinical characteristics and scales	Age 90+ (*n* = 85)	Age < 90 (*n* = 120)	*p*
Age, median (range)	94 (90–103)	83 (64–89)	–
Female patients, *n* (%)	70 (82.4)	93 (77.5)	0.48
Days in the emergency room, median (range)	2 (0–8)	2 (0–17)	0.82
Days of hospitalization, median (IQR)	12 (10–16)	11 (8.5–13)	<0.001
Cancer, *n* (%)			0.24^
No cancer	75 (88.2)	98 (81.7)	
Breast cancer	7 (8.2)	7 (5.8)	
Colon cancer	1 (1.2)	2 (1.7)	
Other cancers	2 (2.4)	13 (10.8)	
Hypertension, *n* (%)	59 (69.4)	72 (60)	0.19
Post-ischemic heart disease, *n* (%)	5 (5.9)	8 (6.7)	0.99
Atrial fibrillation, *n* (%)	19 (22.4)	9 (7.5)	0.003
Chronic heart failure, *n* (%)	7 (8.2)	0 (0)	0.002
COPD, *n* (%)	2 (2.4)	14 (11.7)	0.016
Diabetes, *n* (%)	4 (4.7)	18 (15)	0.022
Thyroid disease, *n* (%)			0.29^
Hypothyroidism	4 (4.7)	17 (14.2)	
Hyperthyroidism	3 (3.5)	0	
Rheumatoid arthritis, *n* (%)	1 (1.2)	1 (0.8)	0.99
Chronic renal failure, *n* (%)	8 (9.4)	7 (5.8)	0.42
Dementia, *n* (%)	21 (24.7)	29 (24.2)	0.99
Stroke, *n* (%)	7 (8.2)	9 (7.5)	0.99
Transient ischemic attack, *n* (%)	5 (5.9)	9 (7.5)	0.78
Depression, *n* (%)	10 (11.8)	24 (20.0)	0.12
Parkinsonism, *n* (%)	2 (2.4)	5 (4.2)	0.48
Osteoporosis, *n* (%)	21 (24.7)	27 (22.5)	0.71
Previous femur fracture, *n* (%)	10 (11.8)	18 (15.0)	0.51
Previous fracture at other site, *n* (%)	31 (36.9)	41 (35.0)	0.89
Hypoacusia, *n* (%)	13 (15.3)	4 (3.3)	0.002
Low vision, *n* (%)	11 (12.9)	10 (8.4)	0.29
Number of falls in the past year, median (range)	0 (0–5)	0 (0–10)	0.24
*CGA on admission to department*			
Handgrip, mean (SD)	11.6 (4.9) [*n* = 57]	14.3 (6.0) [*n* = 104]	0.0014
Barthel Index, median (IQR)	70 (48–85)	85 (55–100)	<0.001
IADL, median (IQR)	2 (0–4.5)	4 (1–6)	0.003
MNA-SF, median (IQR)	9 (8–12)	11 (9–13) [*n* = 111]	0.0073
CIRS Comorbility Index, median (IQR)	3 (3–4)	3 (2–5)	0.85
CIRS Severity Index, mean (SD)	1.83 (0.34)	1.81 (0.42)	0.56
Frailty Index, mean (SD); range	0.51 (0.16); 0.16–0.78 [*n* = 83]	0.44 (0.21); 0.07–0.83 [*n* = 115]	0.015
*Post-surgical complications*			
Anemia, *n* (%)	78 (91.8)	97 (80.8)	0.029
Delirium, *n* (%)	44 (51.8)	29 (24.2)	<0.001
Acute renal failure, *n* (%)	22 (25.9)	16 (13.5)	0.024
Respiratory complications, *n* (%)	18 (21.2)	23 (19.3)	0.75
Urinary tract infection, *n* (%)	23 (27.1)	16 (13.5)	0.015
Cardiac complications, *n* (%)	21 (24.7)	9 (7.6)	0.001
Bed sores, *n* (%)	15 (17.7)	6 (5.0)	0.003
Days of verticalization, median (IQR)	1 (1–2)	1 (1–1)	0.23
Number of medications, median (IQR)	4 (2–6)	5 (3–6)	0.083
Vitamin D, median (IQR)	8.2 (4–24.8)	8.8 (4–20.8)	0.84

COPD, Chronic Obstructive Pulmonary Disease; IQR, Interquartile Range; SD, Standard Deviation; CGA, Comprehensive Geriatric Assessment; IADL, Instrumental Activities of Daily Living; MNA-SF, Mini Nutritional Assessment short form; and CIRS, Cumulative Illness Rating Scale.

Ultra-old patients had a higher disability [median BI of 70 (IQR 48–85); *p* < 0.001] and [median IADL of 2 (IQR 0–4.5); *p* < 0.003], increased risk of malnutrition [median MNA-SF of 9 (IQR 8–12); *p* < 0.01], severe sarcopenia [mean HG of 11.6 kg (SD 4.9); *p* < 0.01] and advanced frailty status [mean FI of 0.51 (SD 0.16); *p* < 0.05]. In addition, these patients were more likely to have atrial fibrillation (22.4%; *p* < 0.01), chronic heart failure (8.2%; <0.01), and hypoacusia (15.3%; *p* < 0.002).

The length of hospital stay was significantly longer in ultra-old patients [median 12 days (IQR 10–16); *p* < 0.001].

### Post-operative complications

Ultra-old patients had an increased incidence of postoperative complications (Table 1), including anemia (91.8%; *p* < 0.05), delirium (51.8%; *p* < 0.001), acute renal failure (25.9%; *p* < 0.05), urinary tract infection (27.1%; *p* < 0.05), acute respiratory failure (24.7%; *p* < 0.01), and bed sores (17.7%; *p* < 0.01).

### Overall survival

**Figure 1:**
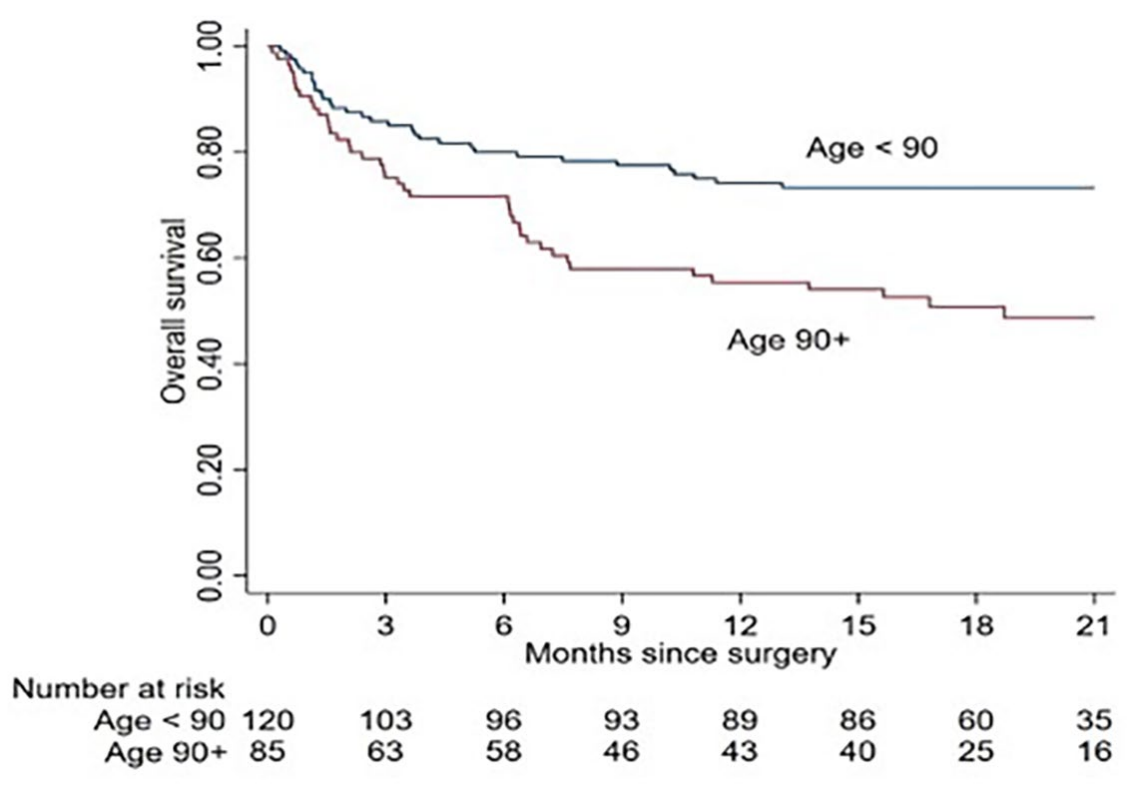
Kaplan-Meier survival curve of ultra-old and control patients after hip fracture.

The median OS for ultra-old patients was 18.7 (7.2–24) months, and, similarly, at 12 months after hip fracture, the OS for ultra-old and younger patients was 55.4% (95% CI: 44.1–65.4) and 74.2% (95% CI: 65.3–81.1), respectively, showing a twofold mortality risk in the ultra-old compared to their younger counterparts (HR = 2.19; 95% CI: 1.38–3.47; *p* = 0.001; Figure 1).

Chronological age did not show any significant association with OS in ultra-old patients compared to the younger control group [univariable Cox regression for OS (all patients): 1.05 (1.02–1.08); *p* = 0.002; ultra-old: 1.06 (0.98–1.16); *p* = 0.12; patients aged 65–89 years: 1.00 (0.95–1.06); *p* = 0.93; P for interaction with age group *p* = 0.22] (Table 2).

**Table 2:** Univariable Cox regression for OS in overall sample and stratified by chronological age.

Clinical characteristics and scales	HR (95% CI); *p*-value			
	All sample (*n* = 205)	Age 90+ (*n* = 85)	Age < 90 (*n* = 120)	P for interaction with age group
Age (1-year)	1.05 (1.02–1.08); *p* = 0.002	1.06 (0.98–1.16); *p* = 0.12	1.00 (0.95–1.06); *p* = 0.93	0.22
Males patients vs. Female patients	1.58 (0.94–2.64); *p* = 0.082	1.90 (0.93–3.88); *p* = 0.079	1.68 (0.79–3.54); *p* = 0.18	0.85
Days from emergency room to surgery	1.13 (1.04–1.22); *p* = 0.005	1.09 (0.89–1.34); *p* = 0.41	1.16 (1.05–1.27); *p* = 0.002	0.53
Cancer (Yes vs. No)	1.45 (0.75–2.39); *p* = 0.33	1.43 (0.60–3.42); *p* = 0.42	1.54 (0.69–3.43); *p* = 0.29	0.85
Hypertension	1.02 (0.63–1.63); *p* = 0.94	0.81 (0.43–1.53); *p* = 0.52	1.12 (0.55–2.28); *p* = 0.76	0.52
Atrial fibrillation	1.35 (0.74–2.46); *p* = 0.33	0.95 (0.47–1.94); *p* = 0.90	1.30 (0.40–4.28); *p* = 0.66	0.65
Diabetes	1.19 (0.59–2.39); *p* = 0.63	0.34 (0.05–2.49); *p* = 0.29	2.22 (1.00–4.95); *p* = 0.05	0.038
Thyroid disease	0.55 (0.24–1.26); *p* = 0.16	0.33 (0.08–1.37); *p* = 0.13	0.84 (0.29–2.39); *p* = 0.74	0.32
Dementia	1.56 (0.95–2.55); *p* = 0.076	1.16 (0.58–2.31); *p* = 0.68	2.18 (1.06–4.46); *p* = 0.033	0.20
Stroke/TIA	1.22 (0.67–2.22); *p* = 0.52	0.83 (0.35–1.97); *p* = 0.67	1.73 (0.75–4.00); *p* = 0.20	0.25
Depression	1.25 (0.70–2.24); *p* = 0.45	0.89 (0.35–2.28); *p* = 0.82	1.85 (0.86–4.00); *p* = 0.12	0.26
Osteoporosis	0.80 (0.45–1.40); *p* = 0.43	0.89 (0.44–1.82); *p* = 0.76	0.63 (0.24–1.64); *p* = 0.35	0.56
Previous femur fracture	0.62 (0.28–1.35); *p* = 0.23	0.81 (0.29–2.28); *p* = 0.69	0.55 (0.17–1.82); *p* = 0.33	0.66
Previous fracture at another site	0.86 (0.54–1.39); *p* = 0.55	0.96 (0.51–1.79); *p* = 0.89	0.74 (0.35–1.56); *p* = 0.43	0.58
Hypoacusia	1.11 (0.51–2.42); *p* = 0.79	0.74 (0.31–1.77); *p* = 0.50	1.01 (0.14–7.37); *p* = 0.99	0.83
Low vision	1.39 (0.69–2.79); *p* = 0.36	1.59 (0.70–3.59); *p* = 0.26	0.78 (0.19–3.26); *p* = 0.73	0.37
Number of falls in the previous year	1.13 (0.99–1.28); *p* = 0.063	1.09 (0.86–1.37); *p* = 0.47	1.15 (0.98–1.34); *p* = 0.085	0.64
*Post-surgery*				
Anemia	1.39 (0.69–2.80); *p* = 0.35	0.77 (0.30–1.96); *p* = 0.58	1.75 (0.61–4.98); *p* = 0.30	0.23
Delirium	2.73 (1.73–4.33); *p* < 0.001	2.30 (1.22–4.33); *p* = 0.010	2.43 (1.20–4.92); *p* = 0.014	0.85
Acute renal failure	2.43 (1.49–3.98); *p* < 0.001	2.19 (1.18–4.09); *p* = 0.014	2.04 (0.88–4.72); *p* = 0.096	0.95
Respiratory complications	2.62 (1.61–4.27); *p* < 0.001	2.92 (1.52–5.61); *p* = 0.001	2.48 (1.17–5.25); *p* = 0.017	0.83
Urinary tract infection	1.19 (0.69–2.07); *p* = 0.54	1.04 (0.53–2.03); *p* = 0.91	0.92 (0.32–2.62); *p* = 0.88	0.87
Cardiac complications	1.74 (1.01–3.00); *p* = 0.045	1.45 (0.76–2.76); *p* = 0.26	1.20 (0.36–3.93); *p* = 0.77	0.81
Bed sores	1.84 (0.99–3.42); *p* = 0.054	1.89 (0.94–3.80); *p* = 0.074	0.60 (0.08–4.39); *p* = 0.61	0.23
Days of verticalization (2+ *vs* 0–1)	2.08 (1.31–3.31); *p* = 0.002	1.84 (1.00–3.38); *p* = 0.048	1.88 (0.91–3.91); *p* = 0.089	0.94
Vitamin D	0.97 (0.95–0.99); *p* = 0.022	0.97 (0.94–0.99); *p* = 0.032	0.98 (0.95–1.01); *p* = 0.22	0.64
*Scales*				
Handgrip (1-point)	0.95 (0.90–1.01); *p* = 0.091	0.97 (0.88–1.06); *p* = 0.51	0.96 (0.89–1.03); *p* = 0.28	0.88
Barthel Index (1-point)	0.98 (0.97–0.99); *p* < 0.001	0.99 (0.98–1.00); *p* = 0.059	0.97 (0.96–0.99); *p* < 0.001	0.091
ADL (1-point)	0.79 (0.71–0.89); *p* < 0.001	0.88 (0.74–1.03); *p* = 0.12	0.74 (0.63–0.88); *p* < 0.001	0.13
IADL (1-point)	0.79 (0.72–0.87); p < 0.001	0.84 (0.74–0.97); *p* = 0.015	0.77 (0.67–0.88); *p* < 0.001	0.29
MNA (1-point)	0.87 (0.80–0.94); *p* = 0.001	0.87 (0.79–0.97); *p* = 0.012	0.90 (0.79–1.02); *p* = 0.11	0.77
CIRS com (1-point)	1.14 (1.03–1.26); *p* = 0.009	1.20 (1.03–1.40); *p* = 0.021	1.14 (0.99–1.31); *p* = 0.076	0.66
CIRS severity (1-point)	2.73 (1.57–4.76); *p* < 0.001	2.76 (1.23–6.23); *p* = 0.014	2.92 (1.31–6.50); *p* = 0.009	0.81
Rockwood (0.1 points)	1.36 (1.19–1.55); *p* < 0.001	1.27 (1.04–1.55); *p* = 0.017	1.39 (1.16–1.68); *p* = 0.001	0.46

Moreover, delirium, acute renal failure, acute respiratory failure, low level of vitamin D, days of verticalization, length of hospital stay, the total time from the emergency room to the hospital orthogeriatric ward, CIRS, Rockwood 40-item score, IADL, and MNA-SF were significant clinical variables at univariable analysis and were selected to enter the multivariate regression analysis.

### Multivariable analysis and prognostic score in ultra-old patients

In the multivariable analysis, malnutrition (MNA-SF; *p* = 0.004), post-operative respiratory acute failure (*p* = 0.004), and delayed time to verticalization (*p* = 0.048) were the main clinical determinants of OS in ultra-old patients (Table 3).

**Table 3:** Multivariable analysis of OS for ultra-old patients and prognostic score development based on internal bootstrap validation.

Clinical variables	HR (95% CI); *p*-value
*Post-surgery*	
**Acute respiratory failure** (Presence vs. absence)	2.69 (1.34–5.00); *p* = 0.004
**Days of verticalization** (2 days+ vs. 0–1 days)	1.88 (1.01–3.40); *p* = 0.048
*Tools*	
**MNA-SF** (1-point)	0.88 (0.80–0.96); *p* = 0.004
*Discriminatory ability*	
**c-index**	0.704 (95% CI: 0.635–0.766)
Prognostic score	HR (95% CI); *p*-value
0 (any clinical variable at the reference level)	1.00 (ref)
3 (2+ Days of verticalization or MNA-SF < 8)	2.52 (1.42–4.49); *p* = 0.002
4+ (Post operative acute respiratory failure and MNA-SF < 8 and 2+ days to verticalization)	3.59 (2.03–6.36); *p* < 0.001

Based on the multiple regression analysis, a prognostic score with internal bootstrap validation was built (Table 3). Namely, a prognostic score of 3 based on delayed verticalization days or malnutrition (MNA-SF <8) was predictive of an intermediate risk of reduced OS in ultra-old patients, and, similarly, a prognostic score of 4 based on the presence of postoperative acute respiratory failure and malnutrition (MNA-SF <8) and delayed verticalization showed the highest predictive risk of reduced OS in ultra-old patients.

The prediction of 1-year mortality was also investigated using multivariate logistic regression, which confirmed postoperative acute respiratory failure (*p* = 0.039) as a significant predictive factor. As a result, no multicomponent prognostic score was developed for 1-year mortality risk.

## Discussion

Hip fracture is a major geriatric injury in old age, and the ultra-old are the fastest-growing group in the aging population, raising additional clinical concerns in terms of functional reserve and recovery after such surgical stress (3). Current evidence is largely insufficient to identify chronological age as a major determinant of mortality and unfavorable outcomes in these patients (3). However, the lack of reliable frailty stratification and the misinterpretation of the statistical odd ratio seem to inflate the true risk, and, to date, there is a growing need to understand whether age alone may drive poorer surgical outcomes and reduced functional benefits in such extremely aged populations.

Notably, our findings contributed to the identification of an ultra-old clinical phenotype. Ultra-old patients were indeed more likely to be female, to have worse functional status, an increased risk of sarcopenia and malnutrition (12, 13), and a higher prevalence of chronic heart failure, atrial fibrillation (14), and hypoacusia. In particular, an overall advanced frailty status, based on a robust methodological stratification, was observed, which may be of key relevance for the clinical understanding of postoperative complications and long-term mortality (15).

To date, some research has highlighted that both frailty and multimorbidity may predict unfavorable clinical outcomes in these ultra-old patients (16), but the lack of a standardized frailty assessment makes the identification of fracture-related outcomes in this at-risk subgroup uncertain (17).

Lunde et al. (18) have previously found that the status of pre-fracture comorbidity is associated with short-term absolute excess mortality and long-term relative excess mortality. Namely, the mortality risk increased along with the Charlson comorbidity index (CCI score) in a large Norwegian nationwide matched population-based cohort study of women aged 55 to 90 years. The absolute excess risk was observed during the first year of follow-up, and thereafter, no excess risk due to the additive interaction was found. In contrast to this study, Vestergaard et al. (19) found little association between pre-fracture comorbidity and excess hip fracture mortality in a large population-based Danish study, attributing 70% of deaths to complications occurring in the first 30 days after fracture and long-term excess mortality to the hip fracture *per se*. However, in patients older than 85, the role of comorbidity was mainly undetermined, and chronological age seems to increase the overall mortality risk.

To the best of our knowledge, this is one of the few studies to systematically assess frailty in ultra-old hip fracture patients, outlining a doubled risk of reduced OS compared to their younger counterparts in long-term observation. In line with this, Schuijt et al. (20) have recently observed that the Rockwood frailty index can be used to guide medical decisions, goals of care, and the benefits of intensive rehabilitation in ultra-old patients after hip fracture. This frailty index was able to predict 90-day mortality and hospital discharge, increasing awareness that measuring frailty in these vulnerable patients may be of key relevance in predicting personalized pathways and treatment decisions.

In the authors’ opinion, the Rockwood frailty scale offers an insight into multiple systems and cumulative clinical deficits through its objective measurements, meaning that it can capture the dynamic change of frailty that is particularly important in relation to hip fracture, physiological reserve, and recovery over time. Indeed, it is a useful tool in understanding how ultra-old patients may maintain resilience to stressors such as hip fractures.

To date, the dynamic nature of frailty in the ultra-old and its predictive value for adverse outcomes are poorly validated, and our findings may initially help understand that frailty may shape long-term mortality by reducing functional reserve due to treatment intensity, rehabilitation, and post-operative complications. Notably, our results also lend support to the fact that chronological age itself is an inaccurate driver of reduced OS. Similarly, Jorissen et al. (21) showed that two-year survival was significantly lower in the ultra-old with higher frailty status after hip fracture, irrespective of chronological age, highlighting the need for frailty prevention measures and appropriate clinical interventions.

It could be hypothesized that frailty is a predisposing factor that, in the presence of stressors such as hip fracture and a series of precipitating factors, may count for an acceleration of the individual’s clinical trajectory, leading to worse outcomes. In our study, we identified malnutrition, delayed mobilization (> 2 days), and postoperative acute respiratory failure as significant determinants associated with decreased OS. Some studies have previously reported the impact of respiratory tract infection as a major factor for immediate mortality (6), and a delayed time between surgery and verticalization was also associated with reduced short-term survival in very old patients after a hip fracture (3). Moreover, malnutrition after hip fracture was observed to predict a higher rate of postoperative complications and increased mortality in patients older than 90 years (13).

Furthermore, the prediction of 1-year mortality confirmed that postoperative acute respiratory failure (*p* = 0.039) was a significant predictive factor irrespective of chronological age. This is in line with de Groot et al. (12) who have reported that nonagenarians had 26.5% higher 1-year mortality than younger counterparts, and dependency, dementia, two or more comorbidities, an ASA score > 3, delayed surgery, and post-operative pneumonia or exacerbation of heart failure were the main determinants of reduced survival.

This study has some limitations owing to the limited sample size, which may inflate the reduced role of chronological age as a determinant of mortality due to the small age difference between the two compared groups. Furthermore, the single-center analysis and missing perioperative data, such as type of surgery and perisurgical parameters, may potentially bias the generalizability of the data and/or any causal assumptions. The effect of the frailty index on long-term functional outcomes and quality of life remains unclear.

However, the strengths of the study are the systematic and rigorous assessment of the frailty phenotype in a selected population of ultra-old hospitalized hip fracture patients and the multi-component prognostic determinants of reduced OS that predict long-term mortality.

## Conclusion

The findings of this study are in line with the stated hypothesis that ultra-old patients differ from other age groups in terms of clinical phenotypes, rates and types of complications, and long-term mortality. Although the higher mortality rate in these patients may be related to their *a priori* lower life expectancy, chronological age alone is an insufficient prognostic determinant of unfavorable outcomes. Whether ultra-old hip fracture patients need a different orthogeriatric treatment strategy is still a matter of debate. However, our multicomponent prognostic score can be used in combination for frailty stratification in ultra-old patients, for timely screening and goals of care discussions prior to surgery that take palliative care into account, and for the potential for new orthogeriatric pathways for the improvement of the appropriateness of care and treatment intensity.

## Data availability statement

The raw data supporting the conclusions of this article will be made available by the authors, without undue reservation.

## Ethics statement

The studies involving human participants were reviewed and approved by Ligurian County ethical Committe IRCCS Policlinico Hospital San Martino, Genoa, Italy. The patients/participants provided their written informed consent to participate in this study.

## Author contributions

DT, CG, MP, and FM: conceptualization. DT, LT, and FM: validation. DT and AS: formal analysis. DT: data curation. DT, LT, AN, and FM: writing—original draft preparation, writing—review and editing. FM: supervision. All authors contributed to the article and approved the submitted version.

## Conflict of interest

The authors declare that the research was conducted in the absence of any commercial or financial relationships that could be construed as a potential conflict of interest.

## Publisher’s note

All claims expressed in this article are solely those of the authors and do not necessarily represent those of their affiliated organizations, or those of the publisher, the editors and the reviewers. Any product that may be evaluated in this article, or claim that may be made by its manufacturer, is not guaranteed or endorsed by the publisher.
