# Supplementary material for: Ultra-old patients and long-term survival after hip fracture: a real-world assessment
Source: Front Med (Lausanne). 2023 Jul 24;10:1200007. doi: 10.3389/fmed.2023.1200007 (PMC10406208; doi:10.3389/fmed.2023.1200007)
Supplement: Supplementary file 1 [file Data_Sheet_1.docx]

Supplementary Material

**Ultra-old patients and long-term survival after hip fracture: a real world assessment**

Debora Tiso^1,2^, Monica Pizzonia^1^, Chiara Giannotti^1^, Luca Tagliafico^1,2^, Alessio Signori^3^, Alessio Nencioni^1,2^, Fiammetta Monacelli^1, 2^*****

^1^Geriatrics Clinic, Department of Internal Medicine and Medical Specialties (DIMI), University of Genoa, 16132 Genoa, Italy.

^2^IRCCS Policlinico San Martino Hospital, Genoa, Italy.

^3^DISSAL, Department of Health Science, University of Genoa, 16132, Via Pastore 16132 Genoa, Italy.

*** Correspondence:**

Fiammetta Monacelli

Associate Professor in Geriatrics

Department of Internal Medicine and Medical Specialties, University of Genoa, Genoa, Italy.

Email: [fiammetta.monacelli@unige.it](mailto:fiammetta.monacelli@unige.it)

Phone: +390105551055

S1  **Comprehensive Geriatric Assessment (CGA)**

S1.1 The Barthel Index


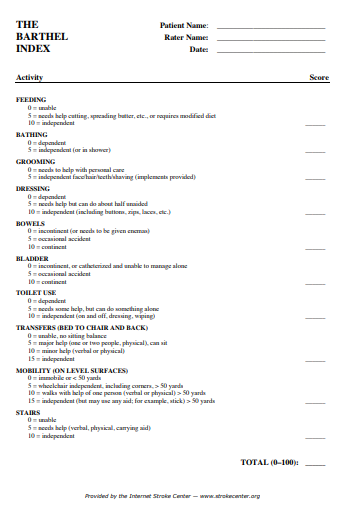


MAHONEY FI, BARTHEL DW. FUNCTIONAL EVALUATION: THE BARTHEL INDEX. Md State Med J. 1965 Feb;14:61-5.

S 1.2 Instrumental Activities of Daily Living (IADL) Scale


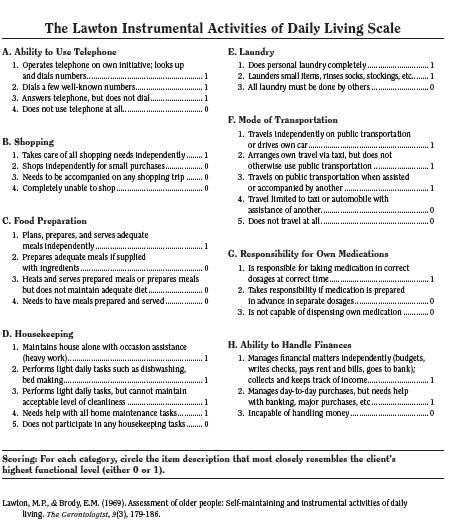


Katz S. Assessing self-maintenance: activities of daily living, mobility, and instrumental activities of daily living. J Am Geriatr Soc. 1983 Dec;31(12):721-7. doi: 10.1111/j.1532-5415.1983.tb03391.x.

S 1.3 Mini Nutritional Assessment- Short Form (MNA-SF)


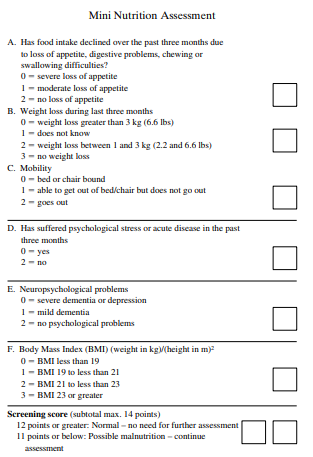


Rubenstein LZ, Harker JO, Salvà A, Guigoz Y, Vellas B. Screening for undernutrition in geriatric practice: developing the short-form mini-nutritional assessment (MNA-SF). J Gerontol A Biol Sci Med Sci. 2001 Jun;56(6):M366-72. doi: 10.1093/gerona/56.6.m366.

S1.4 - Cumulative Illness Rating Scale (CIRS)

**
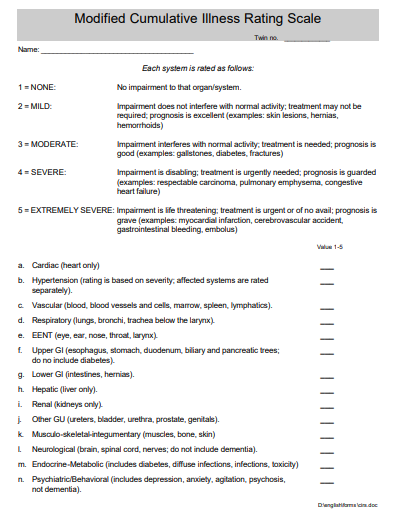
**

Linn BS, Linn MW, Gurel L. Cumulative illness rating scale. J Am Geriatr Soc. 1968 May;16(5):622-6. doi: 10.1111/j.1532-5415.1968.tb02103.x.

S 1.5 4AT


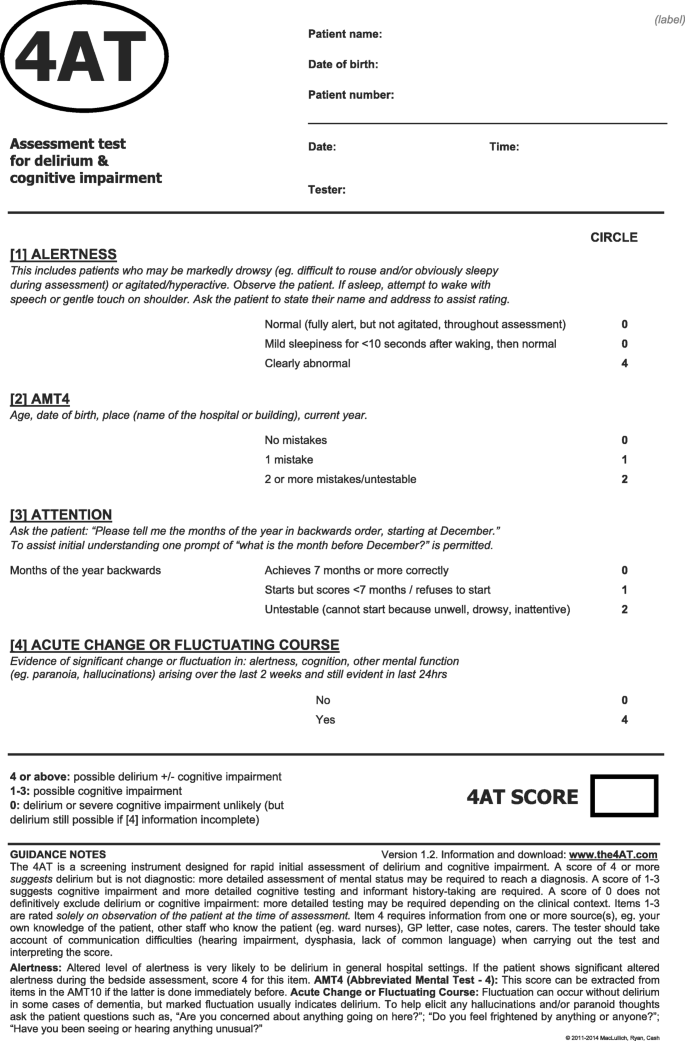


Bellelli G, Morandi A, Davis DH, Mazzola P, Turco R, Gentile S, Ryan T, Cash H, Guerini F, Torpilliesi T, Del Santo F, Trabucchi M, Annoni G, MacLullich AM. Validation of the 4AT, a new instrument for rapid delirium screening: a study in 234 hospitalised older people. Age Ageing. 2014 Jul;43(4):496-502. doi: 10.1093/ageing/afu021. Epub 2014 Mar 2. Erratum in: Age Ageing. 2015 Jan;44(1):175.

S 1.6 Frailty Index according to Rockwood 40-items tool (FI -40 item)
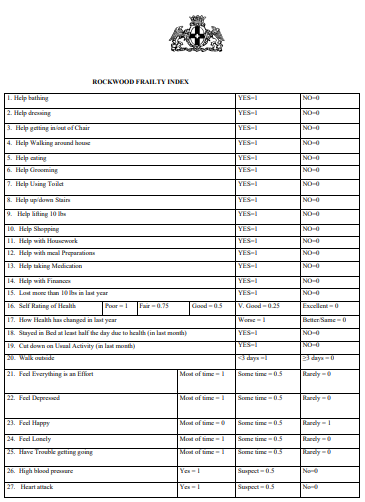

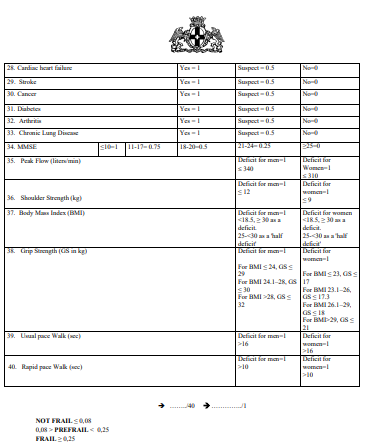


Searle SD, Mitnitski A, Gahbauer EA, Gill TM, Rockwood K. A standard procedure for creating a frailty index. BMC Geriatr. 2008 Sep 30;8:24. doi: 10.1186/1471-2318-8-24.
